# Study Protocol: Transitions in Adolescent Girls (TAG)

**DOI:** 10.3389/fpsyt.2019.01018

**Published:** 2020-02-04

**Authors:** Marjolein E.A. Barendse, Nandita Vijayakumar, Michelle L. Byrne, Jessica E. Flannery, Theresa W. Cheng, John C. Flournoy, Benjamin W. Nelson, Danielle Cosme, Arian Mobasser, Samantha J. Chavez, Lauren Hval, Bernadette Brady, Hanna Nadel, Alison Helzer, Elizabeth A. Shirtcliff, Nicholas B. Allen, Jennifer H. Pfeifer

**Affiliations:** ^1^ Department of Psychology, University of Oregon, Eugene, OR, United States; ^2^ School of Psychology, Deakin University, Burwood, VIC, Australia; ^3^ Department of Psychology, Harvard University, Cambridge, MA, United States; ^4^ Department of Human Development and Family Studies, Iowa State University, Ames, IA, United States; ^5^ Melbourne School of Psychological Sciences, The University of Melbourne, Parkville, VIC, Australia

**Keywords:** adolescence, MRI, puberty, hormones, mental health, longitudinal, social processes, self-development

## Abstract

**Background:**

Despite recent studies linking pubertal processes to brain development, as well as research demonstrating the importance of both pubertal and neurodevelopmental processes for adolescent mental health, there is limited knowledge of the full pathways and mechanisms behind the emergence of mental illnesses such as depression and anxiety disorders in adolescence. The Transitions in Adolescent Girls (TAG) study aims to understand the complex relationships between pubertal development, brain structure and connectivity, the behavioral and neural correlates of social and self-perception processes, and adolescent mental health in female adolescents.

**Methods:**

The TAG study includes 174 female adolescents aged 10.0 to 13.0 years, recruited from the local community in Lane County, Oregon, USA. The participants, along with a parent/guardian, will complete three waves of assessment over the course of 3 years; the third wave is currently underway. Each wave includes collection of four saliva samples (one per week) and one hair sample for the assessment of hormone levels and immune factors; an MRI session including structural, diffusion, resting-state functional and task-based functional scans; the Kiddie Schedule for Affective Disorders and Schizophrenia (K-SADS), a diagnostic interview on current and lifetime mental health; production of a short self-narrative video; and measurement of height, weight, and waist circumference. The functional MRI tasks include a self-evaluation paradigm and a self-disclosure paradigm. In addition, adolescents and their parents/guardians complete a number of surveys to report on the adolescent's pubertal development, mental health, social environment and life events; adolescents also report on various indices of self-perception and social-emotional functioning.

**Discussion:**

The knowledge gained from this study will include developmental trajectories of pubertal, neurological, and social processes and their roles as mechanisms in predicting emergence of mental illness in female adolescents. This knowledge will help identify modifiable, developmentally specific risk factors as targets for early intervention and prevention efforts.

## Background

Puberty and adolescence represent a time of major change in several domains, including physical, neurological, and social development. These changes are important for adolescents to gain greater independence and develop skills for engagement in peer and romantic relationships. However, the transition into adolescence is also a vulnerable period for mental health, with sharp increases in the incidence of many mental disorders, including depression, anxiety disorders, eating disorders, and substance abuse (1–3).

There is currently only limited knowledge on which mechanisms underlie the increased vulnerability for mental illness, as well as on how to identify those most at risk during this developmental phase. Potential mechanisms include pubertal processes, and also behavioral and neural development related to social processes. Pubertal processes include dramatic increases in levels of adrenal and gonadal hormones, as well as physical changes, both of which unfold in unique ways for male and female adolescents. Research is starting to demonstrate that these pubertal processes are linked with processes of structural and functional brain development, often in sex-specific ways (4–6). Further, individual variation in pubertal processes, such as early timing of puberty, has been strongly related to increased risk for mental illness (7). Therefore, pubertal processes are expected to be predictive of risk for mental illness in part through their effects on brain structural or functional development, although direct assessments of this have been scarce (8).

**Figure 1:**
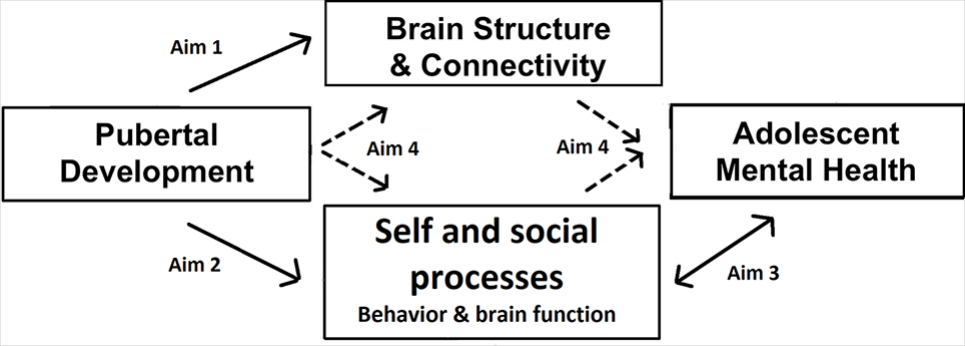
Conceptual model of the study. The arrows indicate the paths examined by each of the specific aims.

At the same time, adolescence is a time of social reorientation and shifts in social behavior, proposed to be mediated by changes in social brain function (9, 10). Understanding of mental states improves and self-evaluation processes change as understanding of the self grows and self-consciousness peaks. Some effects are more pronounced in female adolescents, such as the precipitous declines in self-esteem and increased focus on affiliation/sharing with peers (11–14). These social processes and underlying neural function are crucially related to mental health in adolescence, especially internalizing problems such as depression and anxiety disorders. For example, low self-esteem and high self-consciousness have been associated with risk for internalizing disorders in adolescence (15, 16). More pronounced neural responses to social rejection have been linked to heightened risk for depression (17, 18). Further, co-rumination, excessive negative discussion of personal problems with peers, is predictive of depressive symptoms in adolescents (19). Importantly, some of these social processes have also been linked with pubertal development, such as self-consciousness (20), and the neural responses to social feedback (18) or social self-evaluation (4). Therefore, we propose a pathway from pubertal processes to mental health through the development of social brain function and behavior and brain structural development, as summarized in **Figure 1**.

More knowledge about normative and atypical developmental trajectories and individual differences in the above-mentioned pubertal, neurological, and social processes will help inform prevention and early intervention for mental health issues. It will help to answer key questions about which mechanisms should be targets for prevention and early intervention efforts, as well as which adolescents to focus these efforts on and at which point in development. The Transitions in Adolescent Girls (TAG) study was designed to gain such knowledge, with the broad aim to understand the complex relationships between pubertal development, brain structure and connectivity, the behavioral and neural correlates of social and self-perception processes, and mental health in female adolescents. The study focuses on female adolescents only because pubertal processes, including hormonal and physical changes, differ greatly between the sexes (21). In addition, sex differences have been found in development of social and self-perception processes in adolescence (as highlighted above). Finally, beginning in adolescence, females become at increased risk compared to males for some of the most common mental illnesses, such as depressive and anxiety disorders (22).

The TAG study has four specific aims, as follows.

1. Comprehensively characterize associations within individuals over time in the biological changes associated with puberty. To do so, we will track how indices of pubertal development (hormones, anthropometrics, secondary sex characteristics) covary with changes in task-independent measures of brain development (structure, anatomical connectivity, resting-state functional connectivity).
2. Describe how indices of pubertal change and changes in social cognition and associated brain function also covary over time. To do so, self-evaluation and affiliation will be assessed at both behavioral and neural levels.
3. Examine how changes in social cognition and associated brain function covary with adolescent-emergent mental health problems, in particular symptoms of depression, anxiety, and deliberate self-harm.
4. Test a set of proposed mediation (path) models whereby correlated changes in task-independent brain structure and connectivity, as well as social cognitive brain functioning and behavior, mediate the relationship between pubertal development and associated mental health problems during early adolescence.

## Methods/Design

### Participants

174 female adolescents aged 10.0 to 13.0 years were recruited from the community, together with one of their parents/guardians. See **Table 1** for a list of exclusion and inclusion criteria. Families were recruited primarily through recruitment letters distributed by schools in the greater Eugene/Springfield area (Lane County, Oregon, USA), and to a minimal extent from secure databases of people who registered their interest in our lab's/department's research, recruitment flyers posted around the community or disseminated at community events, and through snow-balling efforts. Recruitment letters were sent to families with children in grade 5 or 6 that were registered as female by the schools. Initially, 189 participants were recruited, but 7 of them failed to meet inclusion/exclusion criteria and another 8 withdrew before completing Time 1 assessments, leading to 174 participants at Time 1 of the study. Parents/guardians have given written informed consent and adolescents assent to participate. Ethics approval was received from Institutional Review Board of the University of Oregon.

**Table 1:** Inclusion and exclusion criteria at time of enrolment.

Inclusion criteria	Exclusion criteria
Female	Diagnosed with a developmental disability
10, 11, or 12 years old	Diagnosed with a psychotic disorder
Fluent in English	Diagnosed with a behavioral disorder, including autism
Normal or corrected-to-normal vision	Taking psychotropic medication other than stimulants
	MRI contraindications^1^
	Report or suspect being pregnant

^1^As described on the Lewis Center for Neuroimaging screening materials, including claustrophobia and presence of ferromagnetic material in the body.

For the final sample of 174 participants, the race and ethnicity distribution was as follows: 66.1% non-Hispanic/Latinx/Chicanx white, 8.6% white Hispanic/Latinx/Chicanx, 0.6% Asian and Hispanic/Latinx/Chicanx, 0.6% African-American and Hispanic/Latinx/Chicanx, 2.9% not further specified Hispanic/Latinx/Chicanx, 0.6% American Indian/Alaskan Native, 0.6% Asian, 0.6% African American, and 19.5% multiracial. This was determined by parent report (reporting on child and both parents, when provided) on a demographic questionnaire and cross-checked with child report during a diagnostic interview (see *Measures* below) when there were possible discrepancies. This distribution shows higher racial and ethnic diversity than in the overall population of Lane County, OR, US; for example, 81.2% of the population was non-Hispanic white and 4.6% were multiracial (23).

One family disclosed that their child identified as non-binary during screening, two other participants self-identified as non-binary during initial data collection, leading to a total of 1.7% reporting a non-binary gender identity at Time 1 of the study; for those three participants, we confirmed that they were assigned female at birth. All remaining participants (98.3%) identified as female.

At Time 1, 1.7% of participating parents/guardians had less than high school education, 13.8% had completed high school or GED, 8.2% had done some college but without a degree, 5.2% had completed trade, technical or vocational training, 18.4% had an associate's degree, 25.3% had a bachelor's degree, 23.0% had a master's, professional or doctoral degree, and 3.4% did not report their education level.

### Timeline and Procedure

This longitudinal study will take place over the course of approximately 3 years, and includes three testing waves. The first wave (T1) took place when participants were 10.0 to 13.0 years old (December 2015–March 2018, *M* age 11.64, *SD =* 0.81), the second wave (T2) took place approximately 18 months after T1 (July 2017–October 2019, *M* age 13.11, *SD =* 0.83), and the third wave (T3; currently underway) will take place approximately 18 months after T2. 163 participants completed T2 (retention rate of 94%), the average time between T1 and T2 was 1.57 years (*SD =* 0.12 years). At each wave participants will complete two laboratory sessions (each 2–3 h in length) 1 month apart, as well as a questionnaire and saliva sampling component at home (see *Measures*). Sessions were on average 33 days apart at T1 and 32 days at T2.

Parents/families who showed interest in the study were provided with information about the project and screened for eligibility over the phone. Those eligible were invited to the University of Oregon's Developmental Social Neuroscience laboratory. At the start of the first session parents/guardians provide written informed consent and children assent to participate. In the first session, participants complete a structured diagnostic interview and part of the questionnaires, and they receive instructions for saliva collection and preparation for the magnetic resonance imaging (MRI) scan (see *Measures* for more details on each component). At the end of the first session, adolescents and their parents/guardians are given the materials to complete saliva samples at home. Adolescents are asked to collect four saliva samples, one per week, between the two lab sessions. About a month later, participants complete the second session, including the MRI scan, the remaining questionnaires, the self-narrative video (*This is me task*), and hair sampling and anthropometric measures (see *Measures* for more details on each component). Procedures described in this paragraph are followed for all three waves, although some questionnaires were added in subsequent waves.

During T1, the following number of participants completed at least part of the following components: diagnostic interview n = 174, questionnaires n = 174, MRI n = 166, home saliva sampling n = 171, hair sampling n = 157. For T2, the numbers were as follows: diagnostic interview n = 163, questionnaires n = 163, MRI n = 143, home saliva sampling n = 152, hair sampling n = 131. The most common reason for missing data on specific components is the participant opting out of that component.

Further, at the time of writing this paper, there are three substudies that participants are invited to take part in but are not part of the original protocol. These three substudies collect data about the gut microbiome; the neural and immune responses to a social self-evaluative stressor; and smartphone usage (passively tracked including usage of apps, geolocation, text and spoken communication, facial expression, sleep information, and physical activity). These are not described here in full because they are not part of the original protocol and they are presented as opportunities for additional research participation to the adolescents and their families.

### Measures

#### Imaging

All scans are acquired using a Siemens Skyra 3.0 Tesla scanner at the Lewis Centre for Neuroimaging at the University of Oregon. Participants go through a mock scanning procedure at every wave, to (re)familiarize them with the scanner and reduce anxiety. At each wave, a T1-weighted structural scan is conducted first, followed by alternating runs of a self-evaluation functional MRI (fMRI) task and a resting-state functional paradigm; two runs of a self-disclosure fMRI task interspersed with a fieldmap; and finally, a diffusion-weighted imaging (DWI) paradigm. See **Table 2** for a summary of the acquisition parameters. Parameters and instructions are the same across all three waves, except for the number of volumes of the resting-state scan, as explained below.

**Table 2:** Scan acquisition parameters.

Scan sequence	voxel size (isometric mm)	number of slices	FOV (mm)	TR (ms)	TE (ms)	Number of volumes	Multiband acceleration factor	Flip angle (°)	Duration (min:sec)
T1-weighted sagittal 3D MPRAGE	1	176	256	2500	3.41	N/A	–	7	5:59
Diffusion- weighted imaging	2	72	208	3920	75.4	2x64 at b = 1000 2 at b = 0	2	90	2x4:54
Resting state BOLD EPI	2.5	60	210	780	32	2x395^1^	6	55	2x5:16
Self-evaluation task BOLD EPI	2	72	208	2000	25	2x180	3 (+ in plane factor of 2)	90	2x6:30
Self-disclosure task BOLD EPI	2	72	208	2000	25	2x225	3 (+ in plane factor of 2)	90	2x8:00
Fieldmap	2	72	208	639	4.37	N/A	–	60	2:16

1 Initially, 270 volumes per run were acquired (duration 3min 38s per run). However, this was increased to 395 volumes per run in August 2017 to increase the chance of acquiring enough high-quality/low-motion volumes (137 participants received the shorter protocol for T1 and four for T2).

##### Structural, DWI, and Resting-State

Participants watch short videos during the structural and diffusion-weighted scans. Participants are asked to close their eyes during the resting-state scan and to not fall asleep (any observations of the participant not following these instructions are noted). Heart rate and breathing are monitored during the resting-state scan using a pulse monitor on the index finger and a breathing monitor across the waist. The sequence for diffusion-weighted images consists of scans in 64 gradient directions at b = 1,000 s/mm^2^, preceded by a non-diffusion-weighted scan (b = 0 s/mm^2^). The whole sequence is repeated with opposite phase encode direction (left-right and right-left) for the purpose of estimating B0 field offsets. Other parameters for this scan, as well as for the T1-weighted structural scan and resting-state fMRI scan can be found in **Table 2**. The only parameter that changed between waves is the number of volumes of the resting-state scan. Initially, 270 volumes per run were acquired. However, this was increased to 395 volumes per run in August 2017 to increase the chance of acquiring enough high-quality/low-motion volumes (137 participants received the shorter protocol for T1 and four for T2).

##### fMRI Self-Evaluation Task

The self-evaluation fMRI paradigm is based on our previous research on self-evaluation in adolescents (4). In this paradigm adolescents will be presented with 50 individual trait adjectives relevant to interpersonal relationships (see **Table 3** for a full list). In the “self-evaluation” condition, participants report whether a given trait describes them, and in the “change” condition participants report whether they believe the trait is something that can change about people in general (i.e., is malleable).

**Table 3:** List of adjectives used in the self-evaluation task.

Prosociality	Social status/sociability^1^	Antisociality/aggressiveness
calm	attractive	aggressive
caring	-awkward	angry
considerate	-boring	assertive
fair	confident	bossy
friendly	cool	controlling
genuine	-depressed	fake
giving	flirty	grumpy
helpful	-insecure	jealous
honest	-lonely	mean
humble	-loner	risky
kind	outgoing	rude
loyal	-plain	selfish
motivated	popular	snobby
nice	-pushover	stubborn
respectful	-shy	
sympathetic	social	
trustworthy	trendy	
welcoming	-ugly	

^1^Items preceded by a hyphen (-) load negatively on the factor.

We selected adjectives based on a pilot sample of N = 100 (after exclusions) female Amazon Mechanical Turk (Mturk) participants, paid $1.50 each, who were required to be age 18–25 years (M = 21.56, SD = 2.0), currently living in the United States, and native English speakers. We asked pilot participants to think about when they were in high school and rate themselves on target adjectives based on how well it described them during that time (6 response options from “Very Poorly” to “Very Well”). We also described two different types of high-status or popular adolescents (populistic, and prosocial-popular) and asked them to rate themselves on each type using the same response options (24). Exploratory factor analysis (EFA) was performed using the psych package (25) in R 3.5.3 (26) using the principal factor method with oblimin rotation. The scree plot indicated a three factor solution was optimal, and items were examined for low loadings or high cross-loadings. Several item exclusions were made, with the final item set determined by a combination of these factors, balanced by the need to retain a sufficient number of items for the self-evaluation task. The final factor solution comprised three factors, one characterized by traits related to social status and sociability (e.g., “popular”, “shy”), a second related to prosociality (e.g., “nice”, “helpful”), and a third related to social aggression or antisociality (e.g., “bossy”, “mean”). Although “prosociality” and “aggression/antisociality” sound like opposites along the same dimension, the factor analysis showed that these were better represented as separable dimensions or factors. Further information on these items can be found in the pilot project repository (https://github.com/jflournoy/svcwords/tree/tag_protocol_paper).

**Figure 2:**
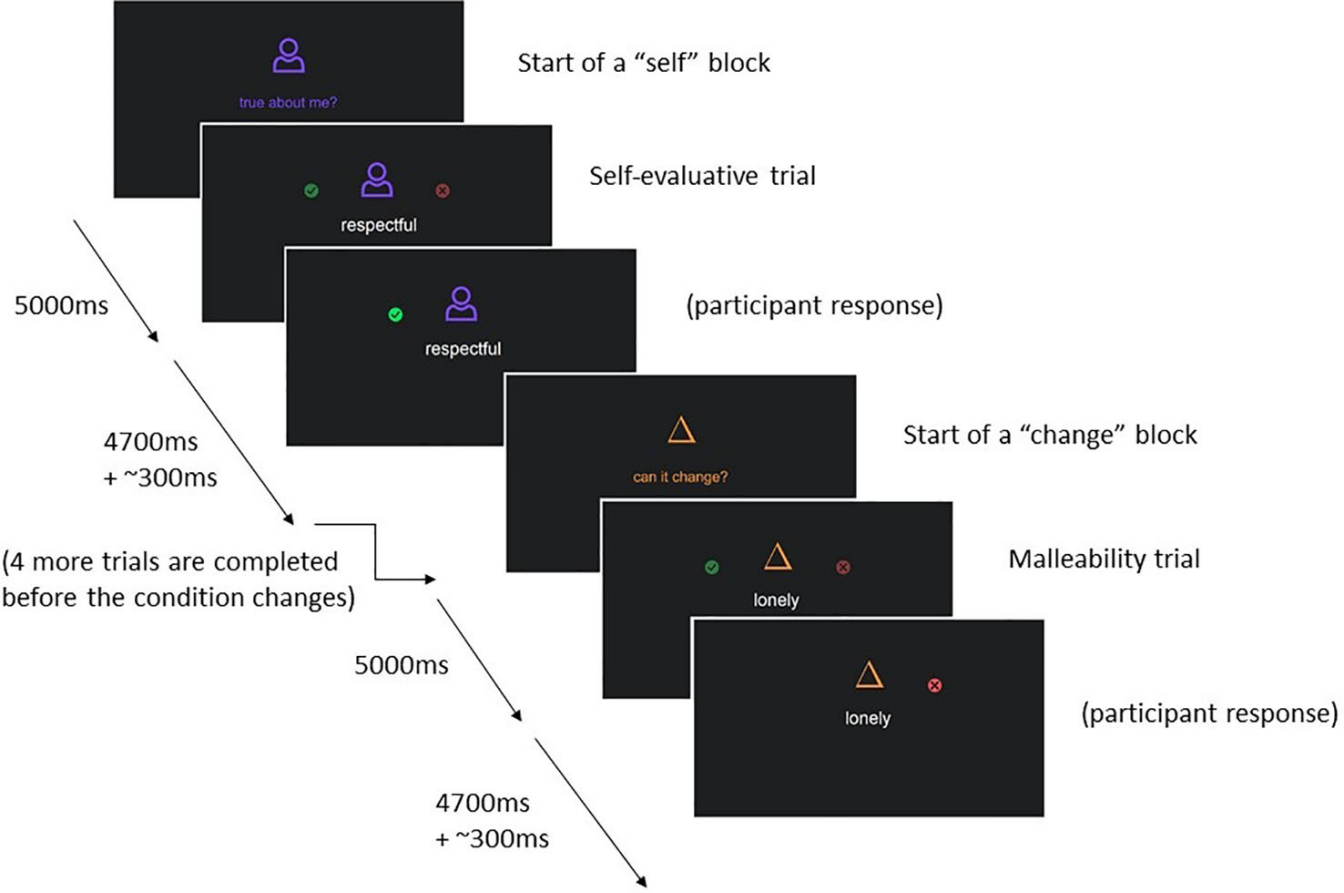
Graphical depiction of the set-up of the self-evaluation fMRI task.

Every trait adjective with corresponding question is presented for 4.7 s, participants can respond any time by pressing a button on a button box, and reaction times are recorded. The task uses a mixed event-related design and is split into two runs. Trait adjectives are nested within alternating “self-evaluation” and “change” blocks. Each block begins with a 5 s instruction cue (self-evaluation or change), followed by five adjectives, each separated by a jittered blank screen presented for approximately 0.3 s; participants complete 10 blocks per run, 5 in each condition. Adjectives that were presented in the change condition in run 1, are presented in the self-evaluation condition in run 2 and vice versa. The order of adjectives is randomized. See **Figure 2** for an illustration of the task design. The code used to present this task and the self-disclosure task can be found online (https://github.com/dsnlab/TAG-fMRI-tasks).

##### fMRI Self-Disclosure Task

In this task, participants agree/disagree with short statements about themselves (presented visually), and subsequently choose either to disclose their answers to their best friend or to keep them private. If participants do not have a best friend they are comfortable sharing with, they are allowed to disclose to a partner or close relative. Half of the items have intimate content (e.g., “sometimes I hide my feelings”, “sometimes I hate going to school”), and the other half have more superficial content (e.g., “sometimes I like wearing makeup”, “sometimes I go to the pool”), see **Table 4** for the full list of stimuli. These stimuli were developed in consultation with a focus group of early adolescent girls. At each trial, the choices to share or keep private are each associated with two to four gold coins, representing two to four pennies. The number of coins presented with each choice option varies from trial to trial. Participants receive the number of pennies associated with the choice they make and are paid their total earnings on the task (up to three dollars) at the end of the session. This is done to calculate an individual's point of subjective equivalence [PSE, calculated as in a similar task from (27)]. The PSE is a measure of how much a person is willing to give up (in case of a negative PSE) or needs to be paid (in case of a positive PSE) to disclose, concretizing the intrinsic value associated with self-disclosure.

**Table 4:** Full list of stimuli of the self-disclosure task.

Superficial	Intimate
braid my hair	act without thinking
burn my toast	can be awkward
can be creative	can be mean
can be messy	can get moody
carry chapstick	can't ignore gossip
decorate my room	can't keep secrets
don't brush my teeth	can't wait to be older
don't make my bed	copy homework
drink milk	count calories
forget people's names	dislike my body
forget to turn off lights	don't like my life
go barefoot	dream about my wedding
go to the movies	feel lonely
go to the pool	feel shy in groups
like burning candles	find homework hard
like cold pizza	get embarrassed
like eating out	get mad at friends
like leftovers	get picked on
like reality TV	get self-conscious
like sleepovers	hate going to school
like to doodle	hide my feelings
like to wear hats	ignore my parents
like watching sports	judge people
like wearing costumes	like breaking rules
like wearing makeup	like flirting
listen to music	make friends easily
paint my nails	pretend to like people
play board games	tease my sibling
read magazines	tell little lies
shower before bed	think I look bomb
sing in the shower	think I'm too quiet
sleep in late	think I'm weird
stay up late	think smoking is gross
take bubble baths	want to be alone
try new foods	want to be popular
try to recycle	weigh myself
use lotion	wish I had more friends
wear bright colors	wish I was in love
wear flip flops	worry about drugs
wear leggings	worry about grades
wear PJs all day	worry about high school
wear sunglasses	worry about kissing

Each statement was preceded by “Sometimes I” (i.e. Sometimes I worry about kissing).

**Figure 3:**
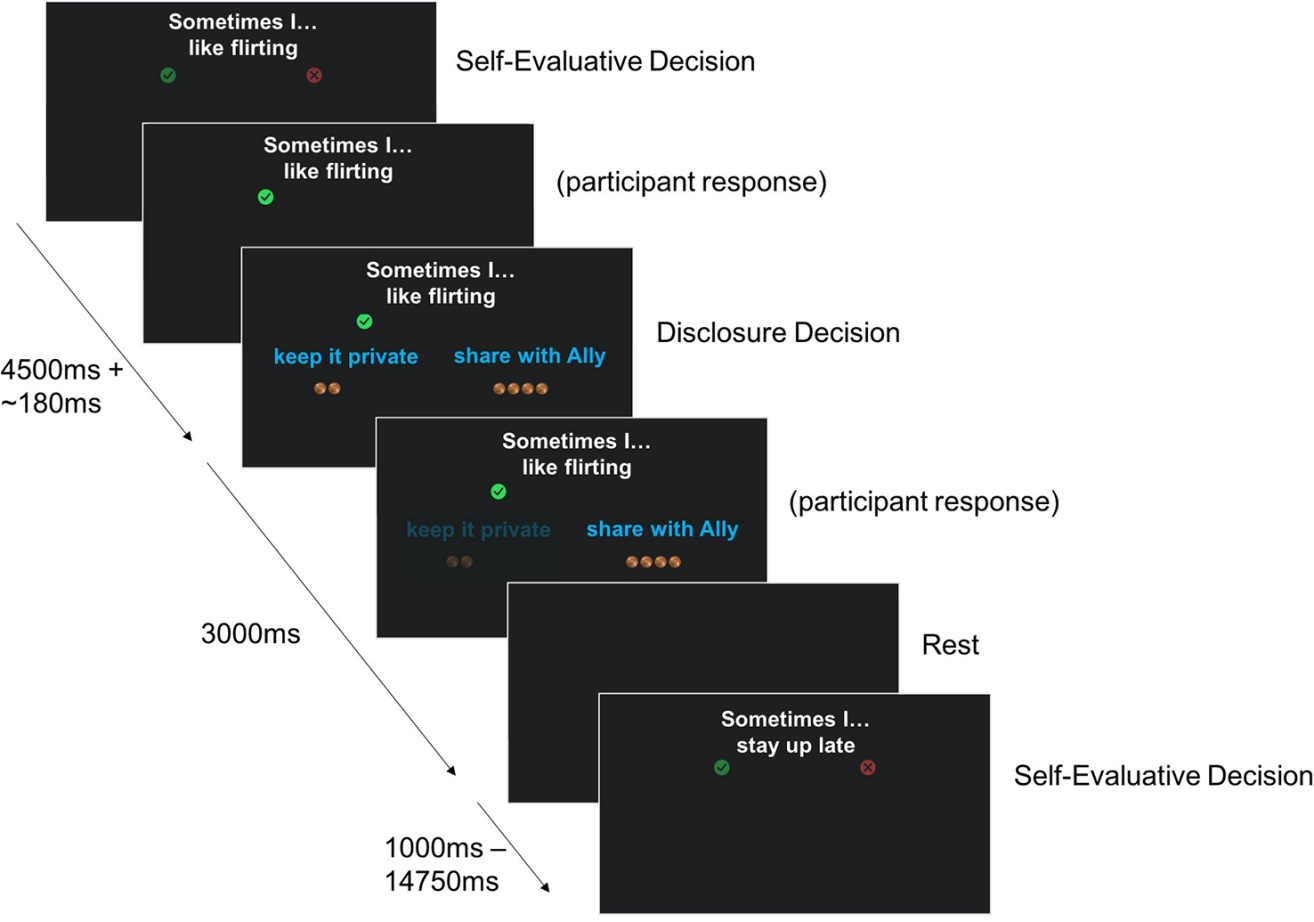
Graphical depiction of the set-up of the self-disclosure fMRI task.

Each statement is presented with the question to agree/disagree for 4.5 s, and with the question about disclosure for 3 s. The evaluative phase was separated from the disclosure phase by an average of 0.18 s, jittered about 0.02 s–0.70 s. A blank screen is shown in between statements for 2.59 s on average (range 1.00 s–14.75 s). Participants' reaction times are recorded. The task is split into 2 runs of 41 statements each. The presentation sequence was optimized to obtain maximal contrast detection between statement depth (“superficial” or “intimate”) and the number of pennies associated with the choices to share versus keep private (gain to share, loss to share or equal value). See **Figure 3** for an illustration of the task design.

At the end of the task, following the MRI scan, participants will be asked to disclose one of the items that they had chosen to share with their friend during the task. The item to disclose will be randomly chosen by the laboratory computer from a subset of items including both intimate and superficial items. Participants will be informed of this ahead of time. The first name of the friend is reported to check for consistency across waves, as well as their age. Finally, participants complete a short survey about the task (the Self-Disclosure Task Experience Survey, see **Supplementary file 1**).

##### This Is Me Task

Participants are asked to make a speech lasting one minute as if they are being interviewed for a new reality TV show. This video task was adapted from the High Risk Social Challenge (28), which is designed to measure social functioning under the stress of social evaluation in adolescents at high risk for developing schizophrenia. In contrast, *This is Me* minimizes the stressful and performative aspects of the speech for use in a typically developing child and adolescent population. Its instructions emphasize that the pretend show is interested in kids of all different backgrounds and types, suggests that participants describe themselves or talk about things that have happened to them, and allows participants up to two minutes to think about what they would like to say before recording. Speeches are videotaped. They are later transcribed for text analysis and coded by trained raters on a subset of items within the social-interpersonal (e.g., social anxiety and engagement) and affective (e.g., facial and nonverbal affect) factors identified from the High Risk Social Challenge ((28); the odd behavior and language factor is excluded and likely exists due to the task origin in a psychiatric context).

#### Diagnostic Interview

The Schedule for Affective Disorders and Schizophrenia for School Aged Children (6–18 Years) Present and Lifetime Version Interview (K-SADS-PL) will be administered as a measure of mental health symptomatology and as an assessment of current and past episodes of psychopathology according to DSM-IV criteria (29). Trained interviewers will administer the semi-structured interview to the adolescent, as well as a shortened interview to the parent/guardian (for report on the adolescent). The parent interview assesses family structure, physical health, medical history, treatment information, family history of mental illness, and autism spectrum disorder symptoms in the adolescent. These items are asked of the parent/guardian because it is expected they will have more information on these topics than the adolescent. The adolescent interview is composed of an introductory interview (including demographic information, school functioning, hobbies, and peer and family relations), and modules assessing present and lifetime history of the following psychiatric disorders: Depression, Mania, Psychosis, Panic Disorder, Agoraphobia, Specific Phobia, Separation Anxiety, Social Phobia, Generalized Anxiety Disorder, Obsessive Compulsive Disorder, Enuresis, Encopresis, Anorexia Nervosa, Bulimia Nervosa, and Post-Traumatic Stress Disorder. Detailed probes and a two- or three-point rating scale with anchors are provided for each criterion, and ratings are made for both current symptoms and symptoms during the most severe past episode. Information regarding the duration and number of episodes for each diagnosis is also collected. At the end of the interview, researchers will also assign a score of general functioning over the past two weeks, based on criteria outlined in the Children's Global Assessment Scale (30). At T1 participants were interviewed about current and lifetime symptoms, but at T2 and T3, they are asked to report only on symptoms since their last interview. Interviewers participate in weekly meetings to ensure adherence to interview administration guidelines, resolve diagnostic dilemmas, and decide on risk management procedures. For T1 20% of interviews were rated twice at the item level (screening items and supplemental items if supplement was completed). Interrater reliability was kappa = .81, which is considered to be in the ‘excellent range’ (29). For T2 and T3 a similar percentage of interviews will be rated twice for interrater reliability estimates.

In addition, self-harm will be examined in detail using an interview-format of the Deliberate Self Harm Inventory [DSHI; (31)] with adolescents. This interview will identify whether adolescents currently or previously engaged in self-harm, as well as the method and extent of engagement. Initial endorsement to intentionally engaging in different types of self-harm will be followed-up by more detailed questions regarding age of onset, frequency, last occurrence, total duration and hospitalization due to the behavior. Note that the DSHI was initially presented in questionnaire format (to the first 125 participants at T1), but this was changed to interview format to improve our ability to conduct risk management in case of self-harm endorsement. The questions remained the same.

#### Questionnaires

Adolescents and parents/guardians are both asked to report on the adolescent's puberty-related physical development, mental health, the family environment, and stressful life experiences. In addition, adolescents complete self-reports on their self-knowledge/self-perception, understanding of mental states, affiliation/self-disclosure, social functioning, and emotion regulation and understanding. See **Table 5** for the full list of questionnaires. All questionnaires are completed on an iPad using Qualtrics and automatically scored, except the Self-disclosure Task Experience Survey, which is completed on paper.

**Table 5:** Questionnaire measures completed by parent/guardian and adolescent by topic.

Category	Reporter	Questionnaire	**T1**	**T2**	**T3**
Puberty	Adolescent	Pubertal Development Scale (PDS) (32)	✔	✔	✔
		Tanner Stage line drawings (33)	✔	✔	✔
	Parent	Parent-reported Pubertal Development Scale (PDS) (32)	✔	✔	✔
Mental health and behavior	Adolescent	Center for Epidemiological Studies-Depression Scale for Children (CES-DC) (34, 35)	✔	✔	✔
		Screen for Anxiety Related Emotional Disorders(SCARED-R) brief version (36)	✔	✔	✔
		Positive and Negative Affect Schedule for Children (PANAS-C) 10-item version (37)	✔	✔	✔
		Cognitive Appraisal of Risky Events - Revised (CARE-R) (38), adapted to focus on social outcome appraisal	✔	✔	✔
		Youth Risk Behavior Survey Middle School Version (YRBS-MS 2015) (39)	✔	✔	✔
		Pittsburgh Sleep Quality Index and Morningness-Eveningness Questionnaire Revised (PSQI-MEQ-R) (40, 41)	✔^1^	✔	✔
		Eating Disorder Inventory (EDI) (42)			✔
		Alcohol Use Disorder Identification Test (AUDIT) (43)			✔
		Cannabis Use Disorder Identification Test (CUDIT) (44)			✔
	Parent	Center for Epidemiological Studies-Depression Scale (CES-D) (45)	✔	✔	✔
		Child Behavior Checklist (CBCL)_(46)	✔	✔	✔
		Social Responsiveness Scale for Parents (SRS-P) (47)	✔	✔	✔
Risk factors and environment	Adolescent	Perceived Stress Scale (PSS) (48)	✔	✔	✔
		Childhood Trauma Questionnaire (CTQ) (49)	✔	✔	✔
		Multidimensional Scale of Perceived Social Support (MSPSS) (50)	✔	✔	✔
		Life Events Questionnaire (51)		✔	✔
		Community ladder [adapted from MacArthur Scale of Subjective Social Status; (52)]		✔	✔
	Parent	Demographics and Socioeconomic Status – updated (DEM-SES v2), see **Supplementary file 2**	✔	✔	✔
		Parenting Stress Index (PSI) short form (53)		✔	✔
		Family Adaptability and Cohesion Evaluation Scale (FACES-III) (54)		✔	✔
		Community ladder [adapted from MacArthur Scale of Subjective Social Status; (52)]		✔	✔
Self-knowledge and self-perception	Adolescent	Self-Perception Profile for Adolescents (SPPA) (55)	✔	✔	✔
		Self-Complexity Scale for Children (SCS-C) (56)	✔	✔	✔
		Revised Self-Consciousness Scale for Children (R-SCS-C) (57)	✔	✔	✔
		Child and Adolescent Mindfulness Measure (CAMM) (58)	✔	✔	✔
		Self-Concept Clarity Scale (SCC) (59)	✔	✔	✔
Understanding mental states	Adolescent	Reading the Mind in the Eyes (RMET) (60)	✔^2^	✔	✔
		Interpersonal Reactivity Index (IRI) (61)	✔	✔	✔
Affiliation and self-disclosure	Adolescent	Shulman Self-Disclosure Scale (SSDS) (62)	✔	✔	✔
		Co-Rumination Questionnaire (CRQ) (63)	✔	✔	✔
		Self-Disclosure Task Experience Survey (SDTES), see **Supplementary file 1**	✔	✔	✔
Social functioning	Adolescent	Intimate Friendship Scale (IFS) (64)	✔	✔	✔
		Big Five Inventory - 10 Item Version (BFI-10) (65)	✔	✔	✔
		Social Achievement Goals Questionnaire (SAQ) (66)	✔	✔	✔
		Social Reward Questionnaire - Child Version (K-SRQ) (67)	✔	✔	✔
		Fundamental Social Motives Inventory (FSMI) (68)			✔
		Relationship and Sexuality questions, see **Supplementary file 3**			✔
		Iowa-Netherlands Comparison Orientation Measure (INCOM) (69)			✔
		Adolescent Social Comparison Scale (ASCS) (70)			✔
Emotion regulation and understanding	Adolescent	Emotion Regulation Questionnaire (ERQ) (71)	✔	✔	✔
		Dweck's Implicit Theories Questionnaire (DIT) (72, 73)	✔	✔	✔
Well-being	Adolescent	EPOCH Scale (74)			✔
		Roberts UCLA Loneliness Scale (RULS-8) (75)			✔
		Satisfaction with Life Scale (76)			✔
		Utrecht Work Engagement Scale for Students (UWES-S) (77)			✔
		Flourishing Scale (78)			✔
Other	Adolescent	Edinburgh Handedness Inventory (EHI) (79)	✔	✔	✔
		Perceived Physical Health [shortened from (80)]		✔	✔
		Physical Activity Habits, see **Supplementary file 4**		✔	✔

^1^The PSQI-MEQ-R was added partway through T1.

^2^At T1, a shorter version of the RMET was administered (inadvertently, only the first half of the questions were included in Qualtrics).

The last three columns indicate if the questionnaire was administered at Time 1, 2, and 3, respectively.

#### Saliva for Hormones

Participants will be asked to collect four saliva samples of 2 ml each at home, in the weeks between the two laboratory sessions. Each sample will be provided through passive drool in the morning, directly after waking, one week apart, on a weekend day. This was done to account for shorter-term variations in the hormones and provide a more stable estimate of basal hormone levels, given that they fluctuate over the day and over the menstrual cycle. This was the preferred method (compared to e.g. sampling at a specific phase of the menstrual cycle) because menstrual cycles tend to be very irregular in early adolescence. Participants are instructed not to eat or brush their teeth before collecting the sample. Families will store the samples in their home freezer and bring it to their second lab session on ice in a cooler bag. Participants and their parents/guardians will be trained on how to collect and store the samples. They will record the time of day at collection and will report on illnesses and medication use in the 24 h before sample collection. Saliva samples are stored in a -80°C freezer in the lab until they are shipped (overnight on dry ice) to the Stress Physiology Investigative Team at the Iowa State University. There they are assayed in duplicate for dehydroepiandrosterone (DHEA), testosterone, and estradiol using Salimetrics Enzyme-Linked Immunosorbent Assay (ELISA) kits. Samples are rerun if the optical density coefficient of variation (CV) is greater than 7% and enough sample is left over to do so. All hormones for each participant are assayed on the same day to minimize freeze-thaw cycles. Moreover, all saliva samples from each participant are assayed on the same plate, to minimize variation in hormone concentrations that may be attributable to plate differences.

#### Saliva for Immune Factors

One additional 1.5 ml saliva sample is collected *via* the passive drool method at session 1 of every wave to examine immune markers. Participants are instructed not to eat or drink anything 30 minutes before collection. Samples are stored at -80°C until they are shipped on dry ice to an external laboratory: the Primate Assay Laboratory, University of California, Davis where samples are assayed in duplicate. Saliva samples are first centrifuged twice at 10,000 g at room temperature (24°C) for 10 min each time to remove cells and mucus, but are not diluted. Samples have a total of one freeze/thaw cycle. A multiplex bead array assay technology (Luminex, Millipore) is used to assay for C-reactive protein (CRP), interleukin(IL)-2, IL-4, IL-6, IL-10, IL-12_p70_, tumor necrosis factor alpha (TNF-α), interferon-gamma (IFN-ɣ) and secretory immunoglobulin A (SIgA) according to manufacturer's instructions.

#### Saliva for Telomere Length

At T1, a 2 ml saliva sample was collected *via* the passive drool method using DNA Genotek Oragene DISCOVER (OGR-500) collection devices during the second lab session to index telomere length. Participants were instructed not to eat, drink, chew gum, or smoke for 30 min before collection. At T2, a saliva sample for telomere length was collected from the first 109 participants completing this wave using the same instructions. Finally, participants who completed a saliva sample for telomere length during T1 will complete another one at T3. Samples are stored at room temperature until shipped for telomere assay by The Blackburn Lab (http://biochemistry2.ucsf.edu/labs/blackburn/) at the University of California San Francisco where samples are assayed in triplicate. This telomere length measurement assay is adapted from the published original method (81, 82). Using this method, the typical average CV is 3%–4%.

#### Hair

At each wave we will collect hair samples to examine DHEA, testosterone, and estradiol levels. Measuring hormones in hair is a relatively new technique, but it has proven to provide a reliable index of longer term levels (83). Five cm long samples will be taken from a 1cm-diameter section of hair (approximately 100 mg of hair), as close to the scalp as possible. Since hair grows about 1cm a month, this will provide an index of the hormone over the past five months. Samples are taken from the posterior vertex of the head, as this has proven to be the most reliable area to measure hormone levels, and it minimizes visibility. Hair samples will be assayed using ELISA in the same lab as the saliva samples.

In addition, participants fill out a brief survey about their hair texture and color; if they dye, bleach, perm, or straighten their hair; how often they blow dry their hair; how often they straighten their hair; and how they have treated their hair (washing, use of products and styling devices) in the days before the lab visit.

#### Anthropometrics

Height, weight, and waist circumference are measured to assess physical development. Measurements will be taken twice at each wave, once at the beginning of the second session and once at the end, to reduce measurement error. Height will be measured using a stadiometer and recorded in centimeters. Weight will be measured using a mechanical column scale with eye level beam and recorded in lbs. Participants are asked to remove shoes, coats and heavy clothing items. Waist circumference will be measured in centimeters using a flexible measuring tape directly on the skin, following the International Society for the Advancement of Kinanthropometry (ISAK) protocols.

#### IQ

Adolescents completed the Wechsler Abbreviated Scale of Intelligence, Second Edition [WASI-II; (84)] as a measure of intelligence. This was administered at T1 only; if for any reason it could not be completed at T1 (N = 19), it will be completed at the next possible wave. The two-subscale version is administered, including Vocabulary and Matrix Reasoning to obtain an estimate of full-scale IQ. This will mainly be used as a control variable in analyses.

### Data Analysis

To determine the sample size for the study, Monte Carlo power analyses were performed in Mplus, with each analysis using 1,000 replications, and an expected attrition rate of 10% per wave. At the time of writing, attrition was only 6.0% between T1 and T2 and 3% between T2 and T3. With the chosen sample size standardized direct effects of 0.235 or larger and moderating effects of 0.15 or larger have at least 80% chance of being statistically significant. For mediation analyses, standardized indirect effects of 0.09 or larger have greater than an 80% chance of statistical significance (assuming the standardized direct effects are at least 0.30).

The majority of data collected as part of the study will be uploaded to the Research Domain Criteria database of the National Institute of Health and will be freely available to other researchers: https://nda.nih.gov/edit_collection.html?id=2315. This includes all imaging data, salivary hormone data, most questionnaires, K-SADS-PL interview data, IQ, and anthropometrics.

Exact procedures used for processing and analysis of the data will depend on the aims and subset of data used in specific empirical papers. Scripts applied to run analyses will be placed on Github: https://github.com/dsnlab/TAG_scripts. However, preprocessing of the imaging data will follow standard pipelines, using fmriprep for fMRI data, freesurfer for structural MRI data, and FSL for diffusion MRI data. The self-evaluation fMRI task will be analyzed as an event-related design, so that both condition (“self” versus “change”) and adjective type can be modeled. The self-disclosure fMRI task will also be analyzed as an event-related design, so that both statement depth (“superficial” versus “intimate”) and choice (to share or keep private) can be modeled. Any region-based analyses will be supplemented with whole-brain or network-based analyses. When conducting null hypothesis significance tests we set our statistical thresholds to at least p < .05 while correcting for multiple comparisons, although the precise approaches to do so might vary between empirical papers as standards in the field continue to evolve (e.g., joint magnitude-extent thresholds for fMRI are now recommended to be set at a minimum of p < .001 for magnitude).

Standardized pipelines have also been developed for the processing of the hormone data. Both salivary and hair samples are assayed in duplicate (see the sections “Saliva for hormones” and “Hair”) and the mean of the duplicates will be used. Salivary hormone concentrations that are non-detectable and too low (i.e., left-censored) will be substituted using the following rules: 1) If other samples from the participant (i.e., from other sampling days) are also not detectable, the means of all samples are replaced with the lower limit of sensitivity (DHEA: 5 pg/ml; T: 1 pg/ml; E2: 0.1 pg/ml). 2) If other samples from the participant are detectable and 50% or more of the remaining samples are below the lower interval of the inter-quartile range (IQR) for the distribution, the mean of the non-detectable sample is replaced with the lower limit of sensitivity for that assay. 3) If other samples from the participant are detectable and less than 50% of the remaining samples are below the IQR for the distribution, the mean of the non-detectable sample is considered missing. For mean salivary hormone concentrations that were too high (i.e., right-censored), the following rules were applied: 1) If 50% or more of the remaining samples are above the upper IQR, the right censored sample is replaced with the upper limit of the standards for that assay (DHEA: 1000 pg/ml; T: 600 pg/ml; E2: 32 pg/ml). 2) If less than 50% of the remaining samples are above the upper IQR, the mean of the right censored sample is considered missing. Hair hormone concentrations (mean of the duplicates) that are non-detectable and too low, are substituted with the lower limit of sensitivity. Hair hormone concentrations that are non-detectable and right-censored are substituted with the upper limit of the standards for that assay. Hormone levels will be log-transformed to correct their non-Gaussian distribution.

General analytic strategies will include latent growth curve models to examine correlated change among the variables of interest (aims 1, 2, and 3) and mediation models (aim 4). The growth curve models will compare linear and quadratic change over time and dual-process growth models will model relationships among variables of interest over time. Mediating effects will be assessed using mediation models appropriate for longitudinal data, which account for confounds such as trait-stability as well as wave-to-wave autocorrelation. To assess significance of indirect (mediated) effects, we will use bootstrap procedures to construct empirical standard errors to account for the skewed sampling distribution of the indirect path estimate. Where relevant and possible, we will examine the impact of demographic variables (such as gender identity or socioeconomic status) on the investigated associations by comparing models with and without these variables.

## Discussion

Despite recent studies linking pubertal processes to brain development (6) and mental health outcomes (7), as well as those demonstrating the importance of brain developmental processes for adolescent mental health (85), there is limited knowledge on the pathways or mechanisms behind the development of mental illnesses such as depression and anxiety disorders in adolescence. The current study will provide a comprehensive picture of the pubertal, neurodevelopmental, and social psychological changes occurring during early-mid adolescence, and their relationship to the emergence of mental health problems, so that modifiable, developmentally specific risk factors can be identified as targets for early intervention and prevention efforts. The study focuses on female adolescents, because of the vast differences between the sexes in pubertal processes, as well as the increased prevalence of internalizing disorders in female adolescents (11).

The current study has several strengths:

- The use of a community-based sample allows for maximum generalizability to the population of female adolescents.
- The longitudinal design with three time points per participant provides more power (compared to the majority of published studies in the field, which have one or two time points), and allows for the exploration of nonlinear trajectories and of developmentally-specific risk predictions.
- The focus on social and self-perception processes such as self-evaluation, affiliation, and understanding of mental states better captures adolescent-specific changes and challenges than for example a focus on basic affective processing.
- The information collected is both comprehensive (e.g., by measurement of pubertal development that includes both hormonal and self-reported data, and by conducting multimodal neuroimaging) and spans several levels (biological, psychological, environmental). Thereby the data is suitable to apply mediation models and examine mechanisms predicting risk for mental illness.

Altogether, this study will help to understand the complex relationships between pubertal development, brain structure and connectivity, the behavioral and neural correlates of social and self-perception processes, and female adolescent mental health. The ultimate goal is to inform novel, developmentally targeted and biologically informed prevention and intervention services that leverage developmental plasticity to help all children, as well as those at higher risk, navigate the transition into and through adolescence with fewer mental health problems.

## Data Availability Statement

The imaging data, salivary hormone data, questionnaires, K-SADS-PL interview data, IQ, and anthropometrics generated as part of this study will be made available through the Research Domain Criteria (RDoC) database: https://nda.nih.gov/edit_collection.html?id=2315. Other data used and/or analyzed during the current study are available from the principal investigator (JP) on reasonable request.

## Ethics Statement

Ethics approval was received from Institutional Review Board of the University of Oregon. Parents/guardians give written informed consent and adolescents assent to participate, and this is repeated at every wave. Research staff have been trained in and follow protocols for confidentiality protection. Following Oregon state law, we have to break this confidentiality in case of non-accidental physical injury to the adolescent, or if we believe a person is in imminent danger of physical harm (including suicide). Risk assessment is done if the participant reports suicidal ideation, self-harm, or information indicating abuse. When risk assessment is done but breaking confidentiality is deemed unnecessary, we provide participants with resources for support.

## Author Contributions

MEAB drafted the manuscript. JP and NA conceptualized the study and wrote the grant funding it. JP, NV, MLB, JEF, TC, JCF, BN, DC, AM, ES, and NA developed the study protocol, including stimulus/paradigm development and identification of surveys. MLB and NA trained K-SADS interviewers. MEAB, NV, MLB, JEF, TC, JCF, BN, DC, AM, SC, LH, BB, HN, and AH collected data. NV, MLB, JEF, TC, JCF, BN, DC, AM, SC, LH, BB, HN, AH, ES, NA, and JP wrote or revised sections of the manuscript. All authors approved the final version of the manuscript.

## Funding

The study described in this manuscript was funded by the National Institute of Mental Health (R01MH107418; PI JP). Author MLB was supported by the National Institute of Mental Health of the National Institutes of Health under Award Number K01MH111951. Author TC was supported by the National Center for Advancing Translational Sciences of the National Institutes of Health under award number TL1TR002371. Author DC was supported by the National Institutes of Health (F31CA232357). The funding agency had no role in the design of the study or the collection, analysis, and interpretation of data or in writing the manuscript, apart from their financial contribution; the content is solely the responsibility of the authors and does not necessarily represent the official views of the National Institutes of Health.

## Conflict of Interest

The authors declare that the research was conducted in the absence of any commercial or financial relationships that could be construed as a potential conflict of interest.
