# Supplementary material for: Study Protocol: Transitions in Adolescent Girls (TAG)
Source: Front Psychiatry. 2020 Feb 4;10:1018. doi: 10.3389/fpsyt.2019.01018 (PMC7010724; doi:10.3389/fpsyt.2019.01018)
Supplement: Supplementary file 1 [file DataSheet_1.pdf]

ID # ____________________

Sharing Task Experience Survey

1. How much did you enjoy playing the Sharing game in the MRI?

1	2	3	4	5
**Not fun at all**	**A little fun**	**Somewhat fun**	**Fun**	**Really fun!**

2. Are you happy to be sharing this information with your friend?

1	2	3	4	5
**Not at all happy**	**A little happy**	**Somewhat happy**	**Quite happy**	**Really happy**

3. Do you feel excited to be sharing this information with your friend?

1	2	3	4	5
**Not at all excited**	**A little excited**	**Somewhat excited**	**Quite excited**	**Really excited**

4. Do you feel nervous about sharing this information with your friend?

1	2	3	4	5
**Not at all nervous**	**A little nervous**	**Somewhat nervous**	**Quite nervous**	**Really nervous**

5. Are you worried about how she will react to your message?

1	2	3	4	5
**Not at all worried**	**A little worried**	**Somewhat worried**	**Quite worried**	**Really worried**

6. How often have you shared similar information with your friend in the past?

1	2	3	4	5
**Never**	**Once**	**Several times in the past**	**Fairly often**	**Very often**
