# Supplementary material for: Study Protocol: Transitions in Adolescent Girls (TAG)
Source: Front Psychiatry. 2020 Feb 4;10:1018. doi: 10.3389/fpsyt.2019.01018 (PMC7010724; doi:10.3389/fpsyt.2019.01018)
Supplement: Supplementary file 2 [file DataSheet_2.pdf]

DEM-SES

The following questions will help us understand your family better. We would appreciate the additional information, but you don't have to answer these questions if you don't want to.

1. How old is your child who is participating in the study today?

2. Does your child have any siblings?
- ❍ Yes (1)
- ❍ No (0)

Display This Question:
If 2. Does your child have any siblings? Yes Is Selected
How many siblings? (1)
What are their ages? (2)
What are their genders? (3)

DEM-SES For questions 3-5, please respond regardless of whether yours is a biological, adoptive, or foster child:

3. What is your child’s ethnic background? (please select ALL that apply)
- ❑ a. Black/ African American (1)
- ❑ b. Hispanic/ Latino/ Chicano (2)
- ❑ c. Native American or Native Alaskan (3)
- ❑ d. White / Caucasian (4)
- ❑ e. Asian (5)
- ❑ f. Native Hawaiian or Pacific Islander (10)
- ❑ g. Multi-racial (6)
- ❑ h. Other (7) ____________________
- ❑ i. Unknown ethnicity (8)
- ❑ j. Decline to respond (9)

4. Concerning the child involved in this study, what is the mother’s ethnic background? (please select ALL that apply)
- ❑ a. Black/ African American (1)
- ❑ b. Hispanic/ Latino/ Chicano (2)
- ❑ c. Native American or Native Alaskan (3)
- ❑ d. White / Caucasian (4)
- ❑ e. Asian (5)
- ❑ f. Native Hawaiian or Pacific Islander (10)
- ❑ g. Multi-racial (6)
- ❑ h. Other (7) ____________________
- ❑ i. Unknown ethnicity (8)
- ❑ j. Decline to respond (9)

5. Concerning the child involved in this study, what is the father’s ethnic background? (select all that apply)
- ❑ a. Black/ African American (1)
- ❑ b. Hispanic/ Latino/ Chicano (2)
- ❑ c. Native American or Native Alaskan (3)
- ❑ d. White / Caucasian (4)
- ❑ e. Asian (5)
- ❑ f. Native Hawaiian or Pacific Islander (10)
- ❑ g. Multi-racial (6)
- ❑ h. Other (7) ____________________

❑ i. Unknown ethnicity (8)
❑ j. Decline to respond (9)

6. What is the level of your education?
❍ a. No schooling completed (1)
❍ b. Elementary school to 8th grade (2)
❍ c. Some high school, no diploma (3)
❍ d. High school diploma or GED (4)
❍ e. Some college credit, no degree (5)
❍ f. Trade, technical or vocational training (6)
❍ g. Associate's degree (7)
❍ h. Bachelor's degree (8)
❍ i. Master's degree (9)
❍ j. Professional degree (10)
❍ k. Doctoral degree (11)

7. Did your education level change during child's life?
❍ yes (1)
❍ no (0)

Display This Question:
If Did your education level change during child's life? yes Is Selected
If yes, what age was your child when you obtained your latest degree (please note additional ages if you have acquired more than one degree over the course of your child's life)

8. If you have a spouse or partner, what is his or her level of education?
❍ a. No schooling completed (1)
❍ b. Elementary school to 8th grade (2)
❍ c. Some high school, no diploma (3)
❍ d. High school diploma or GED (4)
❍ e. Some college credit, no degree (5)
❍ f. Trade, technical or vocational training (6)
❍ g. Associate's degree (7)
❍ h. Bachelor's degree (8)
❍ i. Master's degree (9)
❍ j. Professional degree (10)
❍ k. Doctoral degree (11)

9. If you have a spouse or partner, did their education level change during child's life?
❍ yes (1)
❍ no (0)

Display This Question:
If you have a spouse or partner, did their education level change during child's life? yes Is Selected
If yes, what age was your child when your partner/spouse obtained their latest degree (please note additional ages if they have acquired more than one degree over the course of your child's life)

10. Which of the following best describes your current main daily activities and/or responsibilities?
❍ Working full time (1)
❍ Working part time (2)
❍ Unemployed or laid off (3)
❍ Looking for work (4)

- ❍ Keeping house or raising children full time (5)
- ❍ Retired (6)

11. What is your most recent job title?

12. If you have a spouse/partner, which of the following best describes their current main daily activities and/or responsibilities?
- ❍ Working full time (1)
- ❍ Working part time (2)
- ❍ Unemployed or laid off (3)
- ❍ Looking for work (4)
- ❍ Keeping house or raising children full time (5)
- ❍ Retired (6)

13. If you have a spouse/partner, what is their most recent job title?

14. What is the general level of your annual total household income?
- ❍ a. Up to $25,000 (1)
- ❍ b. $25,000 to 40,000 (2)
- ❍ c. $40,000 to 75,000 (3)
- ❍ d. $75,000 to 100,000 (4)
- ❍ e. Over $100,000 (5)
- ❍ f. Don't know (6)
- ❍ g. Decline to respond (7)

15. Does your child participate in a free-lunch program at school?
- ❍ yes (1)
- ❍ no (0)

16. How many people are currently living in your household, including yourself?

17. How many of the people currently living in your household are:
- children
- adults

of the adults, how many bring income into the household?
