# Supplementary material for: Study Protocol: Transitions in Adolescent Girls (TAG)
Source: Front Psychiatry. 2020 Feb 4;10:1018. doi: 10.3389/fpsyt.2019.01018 (PMC7010724; doi:10.3389/fpsyt.2019.01018)
Supplement: Supplementary file 3 [file DataSheet_3.pdf]

Have you ever had any romantic relationships?

- ○ Yes, currently (1)
- ○ Yes, in the past but not currently (2)
- ○ No (3)

How long have you had your current romantic relationship?

- ○ Less than 1 month (1)
- ○ 1-3 months (2)
- ○ 3-6 months (3)
- ○ 6 months-1 year (4)
- ○ More than 1 year (5)

How many romantic relationships have you ever had?

- ○ 1 (1)
- ○ 2 (2)
- ○ 3 (3)
- ○ 4 (4)
- ○ 5 (5)
- ○ 6 (6)
- ○ 7+ (7)

Do you consider yourself to be:

- ◯ Heterosexual or straight (1)
- ◯ Gay or lesbian (2)
- ◯ Bisexual (3)
- ◯ Questioning or unsure (4)
- ◯ An identify not listed: please specify (5) ____

People are different in their sexual attraction to other people. Which best describes your feelings? Are you:

- ◯ Only attracted to females? (1)
- ◯ Mostly attracted to females? (2)
- ◯ Equally attracted to females and males? (3)
- ◯ Mostly attracted to males? (4)
- ◯ Only attracted to males? (5)
- ◯ Not sure? (6)

What gender do you consider yourself to be?

- ◯ Male (1)
- ◯ Female (2)
- ◯ Questioning or unsure (3)
- ◯ Other identity not listed: please specify (4) ____
