# Supplementary material for: Study Protocol: Transitions in Adolescent Girls (TAG)
Source: Front Psychiatry. 2020 Feb 4;10:1018. doi: 10.3389/fpsyt.2019.01018 (PMC7010724; doi:10.3389/fpsyt.2019.01018)
Supplement: Supplementary file 4 [file DataSheet_4.pdf]

Physical Activity Habits

1) In the last week, on average did you engage in physical activity (30 minutes or more)

0/a week, 1-3/ a week, 3-5/ a week, 6-7/ a week

--On average, would you consider this activity, low, moderate, or high intensity?

2) In the last 3 months, on average did you engage in physical activity (30 minutes or more)

0/a week, 1-3/ a week, 3-5/ a week, 6-7/ a week

-- On average, would you consider this activity, low, moderate, or high intensity?

3) During the school year, on average did you engage in physical activity (30 minutes or more)

0/a week, 1-3/ a week, 3-5/ a week, 6-7/ a week

-- On average, would you consider this activity, low, moderate, or high intensity?
